# Effects of modafinil on non-verbal cognition, task enjoyment and creative thinking in healthy volunteers

**DOI:** 10.1016/j.neuropharm.2012.07.009

**Published:** 2013-01

**Authors:** U. Müller, J.B. Rowe, T. Rittman, C. Lewis, T.W. Robbins, B.J. Sahakian

**Affiliations:** a Department of Psychiatry, University of Cambridge, School of Clinical Medicine, Addenbrooke's Hospital, Cambridge, UK; b MRC/Wellcome Trust Behavioural and Clinical Neuroscience Institute (BCNI), University of Cambridge, Cambridge, UK; c Department of Clinical Neuroscience, University of Cambridge School of Clinical Medicine, Cambridge, UK; d Department of Experimental Psychology, Downing Street, University of Cambridge, Cambridge, UK; e Oxford Uehiro Centre for Practical Ethics, University of Oxford, Oxford, UK

**Keywords:** Modafinil, Executive functions, Memory, Creative thinking, Motivation, Cognitive enhancer

## Abstract

**Background:**

Modafinil, a putative cognitive enhancing drug, has previously been shown to improve performance of healthy volunteers as well as patients with attention deficit disorder and schizophrenia, mainly in tests of executive functions. The aim of this study was to investigate the effects of modafinil on non-verbal cognitive functions in healthy volunteers, with a particular focus on variations of cognitive load, measures of motivational factors and the effects on creative problem-solving.

**Methods:**

A double-blind placebo-controlled parallel design study evaluated the effect of 200 mg of modafinil (*N* = 32) or placebo (*N* = 32) in non-sleep deprived healthy volunteers. Non-verbal tests of divergent and convergent thinking were used to measure creativity. A new measure of task motivation was used, together with more levels of difficulty on neuropsychological tests from the CANTAB battery.

**Results:**

Improvements under modafinil were seen on spatial working memory, planning and decision making at the most difficult levels, as well as visual pattern recognition memory following delay. Subjective ratings of enjoyment of task performance were significantly greater under modafinil compared with placebo, but mood ratings overall were not affected. The effects of modafinil on creativity were inconsistent and did not reach statistical significance.

**Conclusions:**

Modafinil reliably enhanced task enjoyment and performance on several cognitive tests of planning and working memory, but did not improve paired associates learning. The findings confirm that modafinil can enhance aspects of highly demanding cognitive performance in non-sleep deprived individuals.

This article is part of a Special Issue entitled ‘Cognitive Enhancers’.

## Highlights

► Subjective ratings of task performance enjoyment were greater under modafinil. ► Modafinil enhanced performance on cognitive tests of planning and working memory. ► Modafinil can enhance demanding cognitive performance in non-sleep deprived people.

## 1 Introduction

Modafinil (Provigil, 1997) is a wake promoting agent of largely unknown mechanism with demonstrable efficacy in the treatment of daytime sleepiness associated with narcolepsy (Benerjee et al., 2004) and shift-work (Czeisler et al., 2005). Modafinil has been shown to significantly improve performance on tests of executive cognition such as working memory, cognitive flexibility and planning in non sleep-deprived healthy volunteers (Turner et al., 2003; Müller et al., 2004; Minzenberg and Carter, 2008; Finke et al., 2010; Repantis et al., 2010; Mohamed and Sahakian, 2012) and in patients with neuropsychiatric disorders (Turner et al., 2004; Turner, 2006; Minzenberg and Carter, 2008). These pro-cognitive effects of modafinil are of possible therapeutic importance given its low liability for abuse (Deroche-Gamonet et al., 2002), lower risk of adverse effects on the cardiovascular system (Makris et al., 2004; Lynch et al., 2009) and lack of anxiogenic effects that may occur with typical stimulant drugs such as dexamphetamine (Simon et al., 1994).

Turner et al. (2003) originally showed that a single oral dose of modafinil (100 mg or 200 mg) significantly improved performance on tests of digit span, visual recognition memory, visuospatial planning, and stop-signal reaction time (SSRT), but not self-ordered spatial working memory (SWM) in healthy volunteers. The same doses also lengthened response times in tests of decision making, delayed matching to sample, and visuospatial planning, suggesting some effects on speed-error trade-offs. However, other findings are inconsistent with this interpretation, whilst still obtaining reliable cognitive enhancing effects (Müller et al., 2004; Marchant et al., 2009; Winder-Rhodes et al., 2010). Some studies have failed to find robust cognitive enhancing effects on performance of modafinil using similar tests, although some of these were flawed due to insufficient statistical power (see Randall et al., 2005).

In order to address these issues, the present study used a single dose of modafinil 200 mg (Turner et al., 2003; Minzenberg et al., 2008) in a placebo-controlled double-blind design with non sleep-deprived healthy volunteers.

There were three key advances on previous work: First, variations of the cognitive tests which utilised a wider range of cognitive load or task difficulty were employed, in the case of three ‘CANTAB’ tests: self-ordered spatial working memory (SWM); one-touch ‘Stockings of Cambridge’ (SoC) test of planning; and the test of visuospatial paired-associates learning (PAL). Performance improvements in the more difficult task conditions were predicted. Second, we investigated if previously established effects on non-verbal on memory and executive functions could be extended to non-verbal ‘creative’ reasoning, using tasks similar to those adopted in a study of effects of amphetamine by Farah et al. (2009). Finally, we also employed subjective measures of performance, as well as standard analogue mood and cardiovascular indices, because of suggestions that modafinil might influence cognition in part through possible effects on motivation or arousal. Our cognitive tasks were selected so that we could test the hypothesis of cognitive enhancing effects of a single dose of modafinil in healthy participants without sleep deprivation.

## 2 Methods

### 2.1 Participants

Sixty four healthy male (*n* = 31) and female (*n* = 33) volunteers (mean age ± SD = 25.34 ± 3.95, range 19–36 years) were identified via the University of Cambridge Behavioural and Clinical Neuroscience Institute subject panel and via local advertisements. All participants were screened by an experienced psychiatrist (UM) or neurologist (JBR). Subjects were excluded if they had any significant psychiatric history, visual or motor impairment or the concurrent use of any psychotropic medications or any medication contra-indicated with modafinil. In addition, participants with a history of hypertension, cardiac disorders, epilepsy, drug or alcohol abuse were also excluded. All subjects were advised not to consume alcohol or caffeine for 12 h before the testing sessions. All participants were questioned about compliance with alcohol and caffeine restrictions before inclusion into the study. Smoking history was not recorded but as subjects were randomly allocated to the two groups, there should have been no difference between groups. A light breakfast or snack and juice were allowed before, but not during, the experimental session. Each participant gave a written consent prior to testing and received monetary compensation of £25 plus local transport expenses.

### 2.2 Research governance

The protocol was approved by the Cambridge Local Research and Ethics Committee (LREC No. 10/H0305/39) and exempted from clinical trial status by the Medicines and Health Care Products Regulatory Agency (MHRA), London, the national drug licensing agency.

### 2.3 Pharmacological design

This was a randomised, placebo controlled and double-blind study with a parallel group design, deliberately chosen to avoid problems with practice effects that are common in studies with crossover subjects design on tasks of executive and memory functions.

Participants were randomly allocated to one of two blinded medications: modafinil or placebo. This allowed us to control the matching of parallel groups in the course of the study. In order to balance drug conditions for gender, males and females were separately randomised for medications. Unblinding of the medication followed after the data analysis. All volunteers were asked to spend the waiting time with low arousing activities (reading, watching TV or napping) in a day room and were monitored by research nurses. Cognitive testing stated 2 h after drug administration in a silent consultation room at the Wellcome Trust Clinical Research Facility at Addenbrooke's Centre for Clinical Investigation.

### 2.4 Procedure

Subjects completed questionnaires assessing mood and creativity (Visual Analogue Scale, Bond and Lader, 1974; Gough, 1979) and were tested for verbal IQ (National Adult Reading Test, Nelson and Willison, 1991). Following that, a baseline blood pressure and pulse was taken and a single oral dose of 200 of modafinil (Provigil) or placebo (lactose) hidden in identical opaque gelatin capsules was administered with a small glass of water. Dose selection was based on previous cognitive studies in healthy volunteers (Turner et al., 2003) and clinical studies in patients with ADHD (Turner et al., 2004) as well the best recommended therapeutic dose by the British National Formulary 2010 (www.bnf.org). Peak plasma concentrations of modafinil have been obtained 2–3 h after oral administration with an elimination half-life of 10–12 h (Wong et al., 1998; Müller et al., 2004). Therefore, 2 h post-drug administration subjects completed the digit span, a reliable battery of computerised neuropsychological tasks measuring executive function and working memory, and objective creativity and motivational saliency tasks (see Table 1). The test battery was performed in fixed order.

### 2.5 Physiological measures

Blood pressure and pulse measurements were taken using a Criticare Systems Inc. Comfort Cuff (Model 507NJ) at baseline (0 h), during waiting time (+1 h), immediately prior to testing (+2 h), during a short break (+3 h) and after completion of the cognitive test battery (+4 h).

### 2.6 Mood rating and task motivation scale

Participants completed visual analogue scales (VAS, Bond and Lader, 1974) before administration of the drug (baseline) and at intervals during the testing session: immediately prior to testing (2 h post dosing), 1 h into testing (3 h post dosing) and on completion of testing (discharge). At each time point subjects were asked to rate their feeling in terms of 16 dimensions. The measures used in this study were alert–drowsy, calm–excited, strong–feeble, muzzy–clear headed, well coordinated–clumsy, lethargic–energetic, contented–discontented, troubled–tranquil, mentally slow–quick witted, tense–relaxed, attentive–dreamy, incompetent–proficient, happy–sad, antagonistic–amicable, interested–bored and withdrawn–gregarious. The dimensions were presented as 100-mm lines, the two extremes of the emotion (e.g. ‘alert’ and ‘drowsy’) written at each end, and subjects marked where they felt they ranked on each line. Factors of “alertness”, “contentedness”, “calmness” and “tranquility” were calculated as proposed by Bond and Lader (1974) and Herbert et al. (1976).

Task motivation and pleasure was measured using a computerised VAS. After each task participants were asked “Please rate your feelings on the task you took today” and had to slide a pointer accordingly on a scale from “0 = not unpleasant” to “10 = very pleasurable” using a computer mouse.

### 2.7 Neuropsychological measures

Many of the cognitive measures in this study were drawn from the CANTAB battery (www.camcog.com) (Sahakian and Owen, 1992; Robbins et al., 1998), but using novel versions of some of these tasks which included more difficult levels. All computerised tasks were run on an Advantech personal computer (Model PPC-120T-RT), and responses were registered either via the touch-sensitive screen or a response key, depending on the task. A brief description of the key measures for each of the tasks is presented in Table 1. For full details of each outcome measure see CANTABeclipse™ (2011) Test Administration Guide.

To measure non-verbal (visuospatial) declarative memory, we used a version of the CANTAB PAL with an additional level of 12 pattern/location associations (‘Duke no ceiling’, 12 patterns), and an amended version of the Pattern Recognition Memory (PRM) task, which included an additional delayed recognition test after 20 min. For assessment of verbal and non-verbal working memory, we used forward and backward digit span from the Wechsler Adult Intelligence Scale (Wechsler, 1981) and the SWM task from CANTAB with an additional 10-box level. Executive function was tested by a novel variant of CANTAB tower of London task, the ‘one-touch’ version of the Stockings of Cambridge (SOC) spatial planning task (Owen et al., 1995) which included a choice of from one to seven; there were, however, no seven move problems, the most difficult problems were six move.

### 2.8 Statistical analysis

All data were analysed using the Windows versions of SPSS (Version 15, SPSS, Chicago). To investigate the effect of experimental treatment on test performance, differences between group mean performances for single measures were analysed using one-way analysis of variance (ANOVA) or the equivalent non-parametric Kruskal–Wallis ANOVA. Repeated measures ANOVA were used to test the effects of relevant independent within- and between-subjects variables. To clarify the nature of any such differences, planned orthogonal contrasts comparing the effect of modafinil and the effect of placebo were performed where appropriate. All tests employed one-tailed statistics, threshold at *p* < 0.05.

## 3 Results

### 3.1 Demographics

The demographic data are shown in Table 2. The two randomly assigned groups were matched for age, years of education and verbal intelligence (as evaluated with the National Adult Reading Test, NART). For the creativity data, randomization was also successful and resulted in no statistically significant difference between the groups in terms of creativity baseline scores, verbal IQ as indexed by the NART, age, gender and years of education (*p* > 0.05). Furthermore, there was no significant differences between the number of high and low creativity subjects taking modafinil or placebo (*p* > 0.05). The modafinil dose of the study was well tolerated without side effects or complications. One volunteer (*n* = 1 on modafinil) complained about headaches at the end of the testing session. No other adverse events were reported.

### 3.2 Physiological effects – blood pressure and pulse

Physiological readings were taken at five time points during the experiment. Groups were matched on all physiological measures at baseline but the analysis was performed on only the last four physiological measures.

Both systolic and diastolic blood pressure increased over time (*F*(3,135) = 7.4, *p* < 0.001; *F*(3,135) = 9.0, *p* < 0.001, respectively) irrespective of drug treatment Subjects on placebo had higher systolic blood pressure at the end of the study but when compared to modafinil this did not reach significance (*p* = 0.4). There was no effect of drug on diastolic pressure (*p* = 0.8). Similarly, pulse rate increased throughout the experiment (*F*(3,123) = 9.7, *p* = 0.003) but modafinil had no effect (*P* > 0.1).

### 3.3 One touch Stockings of Cambridge (SOC)

**Fig. 1:**
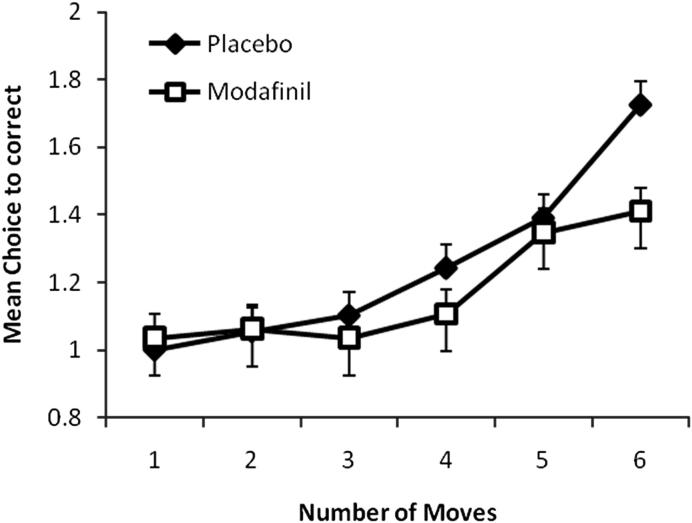
One-touch Stockings of Cambridge (SOC) spatial planning task mean choice to correct. Subjects on modafinil made significantly fewer choices (*p* = 0.002) to achieve the correct answer than those on placebo, particularly at the harder (6 moves). Error bars represent the SEM.

Fig. 1 shows that subjects tested under modafinil made overall significantly fewer attempts to obtain a correct solution relative to the placebo group (main effect drug: drug by difficulty interaction: *F*(6,354) = 2.98, *p* = 0.007). As the figure indicates this improvement by modafinil was especially large at six moves (Table 3).

A mixed repeated measure analysis of variance using the number of moves (1–6) as within subject variables and drug (modafinil vs placebo) as between subject variable was conducted to investigate the effects of latency in the SOC. There was a difficulty effect (*F*(5,305) = 140.5; *p* < 0.001) but no drug × difficulty interaction (*F*(5,305) = 1.3; *p* = 0.259) and no main effect of drug (*F*(1,61) = 0.8; *p* > 0.05). Subjects in both drug conditions took longer to make their responses especially in the more difficult trials.

### 3.4 Digit spans (Table 3)

There were no significant effects of modafinil on digit spans forward or backward (all *p* > 0.1).

### 3.5 Spatial working memory (SWM) (Table 3)

**Fig. 2:**
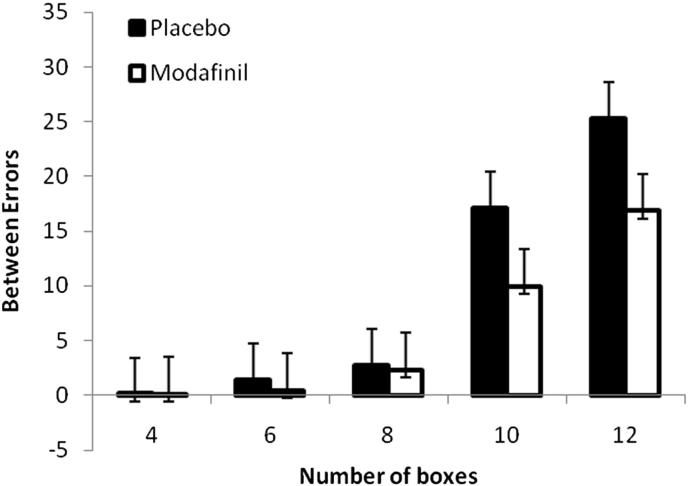
Effect of drug on spatial working memory. Subjects on placebo made significantly more between search errors on the difficult 10-box problems (*p* < 0.05). Error bars represent the SEM.

A two way ANOVA found a significant effect of drug (*F* (1,60) = 7.811, *p* = 0.007) and a drug × difficulty interaction for between errors (*F*(1,60) = 8.320, *p* = 0.005). When analysing the newly introduced 10-box level there was a significant effect of modafinil (*F*(1, 61) = 4.179, *p* = 0.045) with subjects making more errors in the placebo condition (Fig. 2).

### 3.6 Visual pattern recognition memory (PRM) (Table 3)

For the immediate version of the PRM task, a one way ANOVA found no significant between group differences in number of correct visual patterns recognised. Furthermore, no significant differences in latency were identified between groups (*F*(2,57) = 0.48, *p* > 0.05). A one way ANOVA found a significant effect of drug on errors on PRM (*F* (1,58) = 4.6, *p* = 0.036). Subjects receiving modafinil made significantly fewer errors in the delayed PRM than subjects in the placebo group. There were no significant differences in latency between groups (*F*(1,58) = 4.6, *p* > 0.05).

### 3.7 Visuospatial paired associates learning (PAL) (Table 3)

A 2 way mixed ANOVA using group (placebo vs modafinil) as between subject variable and total errors on the PAL shapes (3, 6, 8, 10, 12) as within subject variable was carried out, finding a significant main effect of PAL levels of difficulty on total errors (*F*(4,244) = 45.8, *p* < 0.001). There was no main effect of drug (*F*(1,61) = 91.3, *p* = 0.7) and no drug and difficulty interaction (*F*(4, 244) = 0.5, *p* < 0.71).

### 3.8 Subjective effects (Table 3)

A one way ANOVA found a highly significant effect of modafinil on task motivation (*F*(1,62) = 1131.6, *p* < 0.001). Subjects on modafinil found completing the tasks significantly more pleasurable (mean = 8.9, SD = 0.6) relative to placebo subjects on all tasks except for the Group Embedded Figures task (*F*(1,62) = 0.4, *p* > 0.1).

Subjective measures of contentedness, alertness, tranquillity, and calmness (*p* < 0.001) on the Visual Analogue Scale declined over the session. Subjects in both conditions reported feeling less contended, alert, tranquil and calm. However, there were no significant effects of drug or drug × time interactions on any of the self-reported measures (*p* > 0.1).

### 3.9 Tests of creativity (Table 3)

We found a non-significant trend effect of modafinil on performance in the Group Embedded Figures Task (*F*(1,63) = 3.0, *p* = 0.08), but no significant effect (*p* > 0.1) in the Line Drawing Task.

The effects of modafinil on ATTA flexibility scores were investigated with a one way ANOVA, a main effect of drug (*F*(1,57) = 4.7, *p* = 0.036) was observed. Subjects under modafinil had significantly lower total flexibility scores relative to subjects on placebo. The effect of modafinil on the elaboration scores from the ATTA was investigated with a one way ANOVA and found a trend for main effects of drug on mean elaboration (*F*(1,57) = 3.9, *p* = 0.053) and total elaboration (*F*(1,57) = 3.8, *p* = 0.057). Subjects on modafinil had significantly lower elaboration scores relative to subjects on placebo. Consistent with the above analysis, there was a main effect of drug to reduce mean flexibility scores (*F*(3,55) = 2.9, *p* = 0.045) and a trend effect on mean elaboration scores (*F*(3,55) = 2.8, *p* = 0.053).

## 4 Discussion

Several novel and important findings have arisen from this study. Firstly, consistent with our hypothesis, we demonstrated improvements in performance of non sleep-deprived healthy volunteers with modafinil on certain tests of ‘cold’ cognition such as the CANTAB spatial working memory (SWM), ‘one-touch’ Stockings of Cambridge (SOC) and delayed visual pattern recognition memory (PRM) tasks. Cognitive enhancing effects of modafinil were only seen for the difficult stages of these computerised tasks. Of these, the improvement in CANTAB SWM performance is particularly notable as this is the first time that such an effect of modafinil has been demonstrated in healthy volunteers. There were less errors after modafinil at both the 10- and 12-box level, but this reached significance only for the 10-box level. This finding may be attributed to the use of a new task version with additional 10- and 12-box problems. It is possible that performance was at ceiling level and thus no improvement could be detected in previous studies in healthy volunteers which used an easier version of SWM (Turner et al., 2003) or a version without 10-box problems (Winder-Rhodes et al., 2010).

This study demonstrates for the first time an improvement on CANTAB pattern recognition memory (PRM) delayed recognition. In contrast, no effects were seen on CANTAB paired associates learning (PAL). It is possible that, despite having attempted to make this task more difficult, healthy volunteers still showed ceiling effects. For example, although in this study there was a 12-box version of the task, the total errors for the whole test were only between 18 and 20. In its easier form, this task has been shown to be sensitive in predicting deficits in patients with mild cognitive impairment (Swainson et al., 2001), activating a neural circuitry including the hippocampal formation (de Rover et al., 2011). This failure by modafinil to affect performance on this typical ‘hippocampal’ memory task suggests that the effects of modafinil on cognition in healthy volunteers are limited and may be related to actions on specific neural systems underpinning cognition, such as fronto-striatal circuitry, which are more obviously associated with performance on the Tower of London and spatial working memory tests (Owen et al., 1990). However, the fact that delayed visual recognition (a typical test of temporal lobe function, Owen et al., 1995) did show improvement suggests that we should be cautious in ruling out possible beneficial effects of modafinil on long-term visual memory.

In the study by Turner et al. (2003) modafinil significantly improved performance in a test of inhibitory control (stop-signal reaction time task, SSRT), also subsequently observed for patients with adult attention deficit hyperactivity disorder (Aron et al., 2003). In addition, modafinil significantly slowed performance on the Stockings of Cambridge Task, whilst improving accuracy of the solutions. This improvement was interpreted as resulting from reduced impulsivity; the suppression of over-hastily arrived-at solutions. However, a subsequent study has successfully dissociated the performance improving and slowing effects of modafinil on this task by co-administration of the alpha-1 antagonist prazosin (Winder-Rhodes et al., 2010). The current study, using a more difficult form of the task which included 6 move solutions found no significant effect of modafinil on latency, but the same improvement for the accuracy measure, representing a second replication of this result in our laboratory. Therefore, it appears that modafinil is not simply producing its cognitive enhancing effect through effects on speed-error trade-off, as originally suggested by Turner et al. (2003).

This is the first study investigating the effects of modafinil on measures of non-verbal creativity. It is significant that modafinil did not consistently improve performance on these tests of visuo-spatial or constructive problem solving. It is a limitation of this study that our tests were restricted to non-verbal creativity. Another potential limitation of this study is a sample of relatively high-functioning healthy volunteers, most of them with an academic background.

It is also important to gauge some of the psychological mechanisms by which modafinil may exert its beneficial effects on cognition, both in terms of clinical and shift-work related use. An important finding of this study is that there was a striking increase in task motivation. Participants on modafinil felt considerably more pleasurable after performing individual tasks assessing ‘cold’ cognition and on all but one of the creativity tasks (the Group Embedded Task). This finding is reminiscent of the reinforcing effects of modafinil in humans described by Stoops et al. (2005) which were only evident when there were additional cognitive task demands, suggesting that any motivational effects of the drug derived mainly from its perceived effects on task performance and were thus not similar to those of ‘recreational’ drugs of abuse such as cocaine and amphetamine. The interesting question is whether modafinil enhances motivation through an hypothesised perception by the subject of its ability to enhance performance, or alternatively whether the drug enhances motivational factors which directly impact cognition (and both of these may obtain). It should be noted, however, that modafinil did not produce obvious subjective effects, for example, on arousal, as indicated by visual analogue rating scales or cardiovascular measures.

This finding of motivation enhancing effects of modafinil lends empirical validity to anecdotal evidence from lifestyle use of modafinil that the drug improves concentration and enhances the ability to work for longer periods (Sahakian and Morein-Zamir, 2011). On the other hand, cognitive enhancing effects as described by recreational users of modafinil have to be carefully differentiated from placebo effects. So far, no study has demonstrated cognitive enhancing effects of modafinil in real life situations outside of laboratory settings.

The main finding of this study in healthy volunteers is a clear performance improvement in the most difficult stages of tests of computerised tests of working memory, visual memory and problem solving. These and other published findings suggest that modafinil can improve alertness and motivation and thus potentially reduce apathy and improve functional outcome and adherence to treatment in neuropsychiatric disorders such as substance abuse, depression and schizophrenia (Blackwell et al., 2004; Turner et al., 2004; Martínez-Raga et al., 2008; Morgan et al., 2010; Scoriels et al., 2012, in press). Clinical implications and cognitive enhancing effects outside of lab settings have to be investigated in future research.

## Figures and Tables

**Table 1:** Summary of neuropsychological and creativity battery.

Task	Description	Reference	Important measures
*Working memory*			
Digit spans	A paper–pencil test of verbal memory with immediate recall of digit sequences of increasing length.	Lezak et al., 2004, Wechsler, 1981	Maximal span, forward and backward, total span.
SWM (4, 6, 8, 10, 12 boxes)	Spatial working memory: a computerised test of spatial working memory and strategic search of ‘blue tokens’ hidden in boxes, problems with 4–12 boxes.	www.camcog.com	Total error, between search errors, within search errors, strategy score.
*Planning and decision making*			
One-touch Stockings of Cambridge (SOC)	A computerised test involving planning a sequence of moves to achieve a goal arrangement of coloured balls without moving the balls.	Owen et al., 1995; www.camcog.com	Mean attempts, overall latency.
*Non-verbal declarative memory*			
PRM (Immediate and Delayed)	Pattern Recognition Memory: a computerised dual-choice test of abstract visual pattern recognition with 20 min delay.	Mehta et al., 1999; www.camcog.com	Percentage correct, response latency.
PAL (12 Boxes)	Paired Associates Memory: delayed matching of one to twelve shapes to learnt locations on a touch screen.	Blackwell et al., 2004; www.camcog.com	Total errors, trials to criterion, memory score.
*Non-verbal creative problem solving*			
Group embedded figures task (GEFT)	A nonverbal convergent thinking task requiring subjects to trace a simple shape within a complex figure.	Noppe, 1996; Witkin et al., 2002	Number of correctly identified shapes.
Line drawing task (LD)	Tests of divergent thinking assessing the generation of associations through line drawings.	Wallach and Kogan, 1965; Claridge and McDonald, 2009	Total number of responses and unique responses which no other participant has given.
Abbreviated Torrance task for adults (ATTA)	A task of divergent thinking. Subjects are given a picture-drawing tasks and are asked to draw a picture with a title.	Goff, 2002	Scored for flexibility and elaboration according to the criteria of Goff.

**Table 2:** Mean age, National Adult Reading Test (NART) and formal education for each group. Values shown are the mean and standard deviation of the mean for each group. Age is given in years; NART is the predicted verbal IQ score and education level in years in formal education. ns = not significant (*P* > 0.1).

	Placebo	Modafinil	*F* value	*P* value
Age (years)	24.6 (3.6)	26.2 (4.2)	2.9	ns
NART IQ	124 (3)	122 (5)	3.1	ns
Education (years)	18.6 (2.5)	19.2 (3.1)	0.6	ns
Gough creativity^a^	0.9 (0.2)	0.8 (0.2)	3.2	ns

a transformed Creativity baseline measures.

**Table 3:** Summary of the test results. Values shown for each variable are the mean and standard deviation of the mean for each group. The reported *p* values were derived from one-way and repeated ANOVAs, as appropriate, performed for all two groups.

Task	Placebo mean (SD)	Modafinil mean (SD)	*P* Value
*One touch SOC*			
Mean choice to correct (6 moves)	1.7 (0.4)	1.4 (0.3)	0.009**
*Digit spans*			
Forward span	6.2 (1.0)	6.5 (1.0)	ns
Backward span	4.5 (1.1)	4.7 (1.3)	ns
*SWM*			
Between search errors (10 boxes)	17.2 (16.0)	10.0 (11.3)	0.008**
*PRM*			
Percent correct, immediate	93.7 (10.2)	94.0 (12.1)	ns
Percent correct, delayed	93.8 (10.3)	98.2 (3.5)	0.036*
*PAL*			
Total errors	19.5 (14.6)	18.1 (16.8)	ns
Total trials	10.5 (3.1)	10.2 (3.8)	ns
First memory trial	27.0 (6.2)	27.9 (7.32)	ns
Mean memory to success	3.9 (2.9)	3.6 (3.61)	ns
Mean trial to success	2.1 (0.6)	2.0 (0.7)	ns
Stages completed first trial	2.9 (1.0)	2.3 (1.0)	ns
*Measure of task enjoyment*			
Pleasurable rating of tasks (0–10)	2.3 (0.9)	9.0 (0.6)	<0.001***
*GEFT*			
Creativity score	5.1 (3.4)	6.5 (3.8)	0.08
*LD*			
Creativity score	28.5 (11.8)	30.8 (10.5)	ns
*ATTA*			
Mean elaboration	2.4 (1.1)	1.9 (0.8)	0.053
Mean flexibility	1.2 (0.3)	1.0 (0.3)	0.057

ATTA = Abbreviated Torrance Test for Adults; GET = Group Embedded Figures Task, LD = Line Drawing; PAL = Paired Associate Learning; PRM = Pattern Recognition Memory; SOC = Stockings of Cambridge; SWM = Spatial Working Memory; * = *p* < 0.05, ** = *p* < 0.01, *** = *p* < 0.001. ns = not significant (*p* > 0.1).
